# Fifteen-Year Differences in Indications for Cardiac Resynchronization Therapy in International Guidelines—Insights from the Heart Failure Registries of the European Society of Cardiology

**DOI:** 10.3390/jcm11113236

**Published:** 2022-06-06

**Authors:** Agata Tymińska, Krzysztof Ozierański, Emil Brociek, Agnieszka Kapłon-Cieślicka, Paweł Balsam, Michał Marchel, Maria G. Crespo-Leiro, Aldo P. Maggioni, Jarosław Drożdż, Grzegorz Opolski, Marcin Grabowski

**Affiliations:** 1 First Department of Cardiology, Medical University of Warsaw, 02-091 Warsaw, Poland; agata.tyminska@wum.edu.pl (A.T.); emil.brociek@gmail.com (E.B.); agnieszka.kaplon@gmail.com (A.K.-C.); pawel.balsam@wum.edu.pl (P.B.); michal.marchel@wum.edu.pl (M.M.); grzegorz.opolski@wum.edu.pl (G.O.); marcin.grabowski@wum.edu.pl (M.G.); 2 Unidad de Insuficiencia Cardiaca Avanzada y Trasplante Cardiaco, Hospital Universitario A Coruna, CIBERCV, 15006 La Coruna, Spain; marisacrespo@gmail.com; 3 ANMCO Research Centre, 50121 Florence, Italy; maggioni@anmco.it; 4 EURObservational Research Programme, European Society of Cardiology, Sophia-Antipolis, 06903 Valbonne, France; 5 2nd Department of Cardiology, Central University Hospital, Medical University of Lodz, 92-213 Lodz, Poland; jaroslaw.drozdz@umed.pl

**Keywords:** cardiac resynchronization therapy, heart failure, cardiomyopathy, left bundle branch block

## Abstract

Cardiac resynchronization therapy (CRT) applied to selected patients with heart failure (HF) improves their prognosis. In recent years, eligibility criteria for CRT have regularly changed. This study aimed to investigate the changes in eligibility of real-life HF patients for CRT over the past fifteen years. We reviewed European and North American guidelines from this period and applied them to HF patients from the ESC-HF Pilot and ESC-Long-Term Registries. Taking into consideration the criteria assessed in this study (including all classes of recommendations i.e., class I, IIa and IIb, as well as patients with AF and SR), the 2013 (ESC) guidelines would have qualified the most patients for CRT (266, 18.3%), while the 2015 (ESC) guidelines would have qualified the least (115, 7.9%; *p*-value for differences between all analyzed papers <0.0001). There were only 26 patients (1.8%) who would be eligible for CRT using the class I recommendations across all of the guidelines. These results demonstrate the variability in recommendations for CRT over the years. Moreover, this data indicates underuse of this form of pacing in HF and highlights the need for more studies in order to improve the outcomes of HF patients and further personalize their management.

## 1. Introduction

The incidence and morbidity of heart failure (HF) is increasing, with a high prevalence in developed countries, where it affects approximately 1–2% of adults, rising to ≥10% in those aged 70 years or over. Prognosis of HF patients remains poor, as the 5-year mortality rate after diagnosis is approximately 50% [1,2]. Although there are well established therapies for HF with reduced ejection fraction (HFrEF) further research is necessary to improve the symptoms and outcomes of patients with HF.

CRT (cardiac resynchronization therapy) is a method of treating advanced chronic HF, aiming to improve synchrony of both ventricles [1,3]. In patients who respond to the therapy (“responders”), CRT is an effective treatment for reducing HF morbidity and mortality, as well as improving patients’ quality of life. However, the selection criteria for CRT are still imperfect, and it should be noted that it is not certain that all HF patients benefit from this form of cardiac pacing.

In patients with primarily chronic HF (i.e., excluding patients with primarily high degree atrioventricular block, as this indicates that the patient will need permanent ventricular pacing), the indications for CRT have been changing in line with the publication of new international guidelines i.e., of the European Society of Cardiology (ESC) and American associations (ACC—American College of Cardiology; AHA—American Heart Association; HFSA—Heart Failure Society of America; HRS—Heart Rhythm Society). Over the years, the decisions on CRT implantation were made using, among others, numerous of the following criteria: the presence of a left bundle branch block (LBBB); left ventricular ejection fraction (LVEF); different QRS durations; different levels of severity of symptoms of HF according to the New York Heart Association (NYHA) class; left ventricular dimensions; the presence of atrial fibrillation (AF) or sinus rhythm (SR); indications for permanent conventional pacing; the presence of optimal medical therapy for HF [1,3,4,5,6,7,8,9,10,11]. Furthermore, successive guidelines that are published shortly after the previous ones often substantially change the profile of a patient who is eligible for CRT. Therefore, the current focus of HF therapy is shifting towards better characterization of the underlying etiology of HF and personalized management.

This study aimed to investigate the changes in eligibility for CRT of real-life patients with HF from the ESC-HF registries according to the differences in international guidelines published in the last fifteen-years, with a particular focus on the newest ESC (2021) and ACC/AHA/HFSA (2022) recommendations.

## 2. Patients and Methods

### 2.1. Study Design

The data was collected from the multi-center, prospective, observational ESC-HF Pilot and the ESC-HF Long-Term registries of the European Society of Cardiology (ESC). These registries lasted from 2009 to 2010 in 136 European cardiology centers (including 29 centers from Poland) and from 2011 to 2015 in 211 European cardiology centers (including 35 centers from Poland), respectively. The studies enrolled outpatients and inpatients with chronic, worsening or new-onset HF who were at least 18 years of age and met the diagnostic criteria for HF. There were no other specific exclusion criteria. All participating patients signed informed consent. Records collected in both registries referred to clinical characteristics, laboratory tests’ results, HF management and one-year follow-up. A detailed study design was published previously [12,13]. The study protocol was approved by the local ethics committees.

The ESC-HF Pilot and the ESC-HF Long-Term registries enrolled 5118 and 12,440 patients across Europe, respectively. The current analysis consisted of 1456 (out of 2019 patients enrolled in Polish centers) both ambulatory and hospitalized, clinically stable HF patients. Only patients with available data on CRT eligibility criteria were included. Patients with a paced rhythm on ECG were excluded from the analysis. Criteria for CRT implantation in the major international (ESC and America’s) guidelines from 2007 to 2022 that were considered for this study are listed in the Supplementary Table S1. The CRT eligibility criteria were assessed in the studied group. Common classification of recommendations presented in the guidelines were used: class I (indicated/recommended); class IIa (should be considered); class IIb (may be considered); class III (contraindicated/not recommended). The following parameters were considered in the aforementioned guidelines and analyzed in this study: LVEF, presence of LBBB, QRS duration, SR or AF, the NYHA class, and HF etiology (ischemic). Patients with SR or AF were analyzed separately according to the general indications for CRT. Patients’ clinical status, comorbidities, expected lifetime duration and actual time of HF optimal pharmacotherapy were not considered in the analysis.

Data on patients who presented with indications for pacing, atrioventricular node (AVN) ablation or pacemaker/implantable cardioverter defibrillator (ICD) upgrade to CRT was not available in the database and, therefore, was not considered in the analysis. Indications for CRT-pacemaker (CRT-P) or CRT-defibrillator (CRT-D) were also not the target of the analysis.

### 2.2. Statistical Analysis

The results were presented as median and quartiles for continuous variables, and as frequencies and percentages for ordinal variables and non-normally distributed continuous variables. The frequencies of the categorical variables were compared by Fisher’s exact test. A *p*-value below 0.05 was considered significant for all tests. All tests were two-tailed. Statistical analyses were performed using SPSS software, version 22 (IBM SPSS Statistics 22, IBM, New York, NY, USA).

## 3. Results

### 3.1. Clinical Characteristics

Baseline characteristics of the studied group are presented in Table 1. Briefly, median age was 67 (58–76) years and 67% of the patients were males. Median LVEF was 37% (25–50). LVEF ≤ 35% was observed in most patients (55%). Ischemic etiology of HF was present in 54%. Numerous comorbidities were highly prevalent (i.e., coronary artery disease, hypertension, peripheral artery disease, diabetes, chronic kidney disease, chronic obstructive pulmonary disease, previous stroke, or transient ischemic attack). Use of HF guideline-recommended medications was on a satisfactory level.

### 3.2. Patients Eligible for CRT Implantation

In the study group 1014 (69.6%) patients had SR and 442 (30.4%) patients had AF. When considering patients with SR, the smallest number of patients with class I indications for CRT was 45 (4.4%) following the 2012/2013 (ACC/AHA/HRS), 2021 (ESC) and 2022 (ACC/AHA/HFSA) guidelines, while the biggest number of patients with class I indications for CRT was 137 (13.5%) following the 2010 (ESC) guidelines (*p*-value for differences between all groups < 0.0001). Class IIa indications were present in 17 (1.7%) and 97 patients (9.6%) based on the 2015 (ESC) and 2022 (ACC/AHA/HFSA) guidelines (*p*-value for differences between all groups < 0.0001), respectively. Class IIb indications were present in 28 (2.8%) and 80 patients (7.9%) using the 2015 (ESC) and 2022 (ACC/AHA/HFSA) guidelines, when compared to the 2013 (ESC) guidelines (*p*-value for differences between all groups < 0.0001).

Within the AF cohort, indications for CRT implantation ranged from 17 patients (3.8%) using the 2015 (ESC) and 2016 (ESC) guidelines, to 86 patients (19.5%) based on 2022 (ACC/AHA/HFSA) guidelines (*p*-value for differences between all groups < 0.0001).

Taking into consideration the criteria assessed in this study (including all classes of recommendations and patients with AF and SR), the highest number of patients that would have qualified for CRT was 266 (18.3%) by using the 2013 (ESC) guidelines, while the fewest was 115 (7.9%) when considering the 2015 (ESC) guidelines (*p*-value for differences between all groups < 0.0001). There were only 26 patients (1.8%) who would be eligible for CRT using the class I recommendations across all of the guidelines (the common criteria in these patients were: SR, LVEF ≤ 35%, LBBB, QRS ≥ 150 ms, NYHA class III or ambulatory class IV). The results are presented in Table 2.

The differences in patients’ eligibility for CRT according to the recent (ACC/AHA/HFSA (2022) and ESC (2021)) guidelines are presented in Table 3 and Table 4. Among the 1456 patients in the study group, 256 (17.6%) and 185 (12.7%) patients were eligible for CRT based on the ACC/AHA/HFSA (2022) and ESC (2021) guidelines (*p* < 0.001), respectively (Table 2). The largest difference in eligibility between these guidelines were observed within patients with AF—86 (19.5%) and 31 (7.0%) would have qualified for CRT according to the ACC/AHA/HFSA (2022) and ESC (2021) guidelines (*p* < 0.001), respectively. Whereas, in terms of class I indication for CRT, 45 (4.4%) patients with SR were eligible for CRT according to both guidelines.

## 4. Discussion

This study assessed the applicability of eligibility criteria of real-life patients with HF by comparing the international guidelines for CRT, with special focus on the latest 2021 ESC and 2022 AHA/ACC/HFSA papers [1,3,7]. The criteria described in the guidelines are mostly derived from randomized control trials or meta-analyses which are rigorously interpreted by the guideline task forces forming specific recommendations. In the last fifteen years, as more scientific evidence was published, further guidelines emerged. This study demonstrates areas of consistency and inconsistency in recommendations for CRT and suggests underuse of CRT in the population of HF patients.

**Figure 1:**
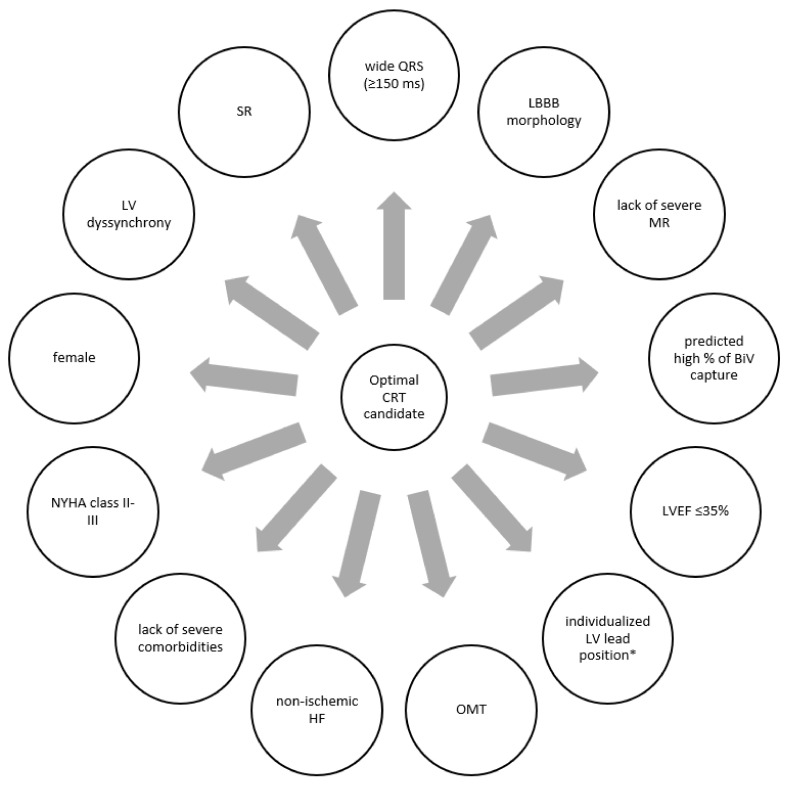
Selected clinical parameters associated with higher probability of favorable response to cardiac resynchronization therapy. BiV—biventricular; CRT—cardiac resynchronization therapy; HF—heart failure; LBBB—left bundle branch block; LV—left ventricle; LVEF—left ventricle ejection fraction; MR—mitral regurgitation; NYHA—New York Heart Association; OMT—optimal medical therapy; SR—sinus rhythm; %—percentage; * posterolateral lead position; correct pacing parameters; avoided scar areas.

CRT increases strength and improves the synchrony of ventricular contractions, extends left ventricular diastolic filling time, increases cardiac output and systolic blood pressure, as well as reduces functional mitral regurgitation [14]. This translates into visible clinical effects: reduction of the severity of HF symptoms, improvement of exercise tolerance, as well as a reduction of morbidity and mortality due to HF [3]. In addition to the clinical efficacy of CRT, its cost-effectiveness has been evaluated in several analyses [15,16,17,18,19,20]. After reviewing literature that describes years of experience with CRT, the optimal profile of a patient who could benefit most from CRT can be specified (Figure 1) [1,3,7,21,22,23,24]. Before evaluating the indications for CRT, the patient should receive optimal treatment of HF according to the current best medical knowledge for at least 3 months (or even 6–9 months) [1,25]. Optimizing the treatment of comorbidities and achieving the highest recommended (tolerated) doses of drugs that improve the prognosis in HF may result in the withdrawal of indications for CRT.

CRT has been shown to be clinically useful mainly for patients with LVEF ≤ 35%, who represented approximately half of the study group. Some studies considered an LVEF ≤ 30% [26,27]. This specific indication for patients with LVEF ≤ 30% was included only in 2012 and 2022 guidelines [6,7]. Interestingly, in this study only 1 patient could have qualified for CRT based on this criterion (SR, ischemic HF, NYHA class I, QRS width ≥ 150 ms and LVEF ≤ 30%).

CRT is most effective for patients with SR, although several trials have shown the benefits of its use for patients with AF [28,29,30]. In these patients, the success of CRT depends primarily on the proportion of achieved biventricular pacing, and for many patients, AVN ablation is required. In this study, in all but the AHA/ACC/HFSA 2022 guidelines, more patients with SR than AF met the criteria for CRT. This is probably due to the fact that in recent years there has been a visible trend towards more intensive heart rate/rhythm control with the use of pharmacotherapy, and especially using invasive methods (AF or AVN ablation) [1,7,31]. In this study, the presence of an uncontrolled heart rate in patients who are potential candidates for AF or AVN ablation were not analyzed due to the lack of specific data.

The beneficial effects of CRT have been extensively proven in patients with NYHA class II–IV HF (over 90% of patients in our study). While one study observed a non-significant trend towards a lower risk of death from any cause in patients with NYHA class I, LBBB, and ischemic cardiomyopathy, this indication was included in the AHA/ACC/HFSA 2022 guidelines, but not in the ESC 2021 guidelines [1,3,7,32,33,34]. Additionally, the current ESC 2021 guidelines do not include HF etiology in the decision-making process for CRT implantation. However, it was shown that patients with ischemic heart disease are at greater risk of sudden cardiac death and, therefore, benefit more from ICD than patients with non-ischemic cardiomyopathy [1,3,35]. On the other hand, extensive myocardial scarring in patients with ischemic HF may attenuate clinical benefit from CRT, and if possible, lead placement should avoid scarred areas [1,3]. NYHA class varies with time, depending on the current condition of the patient, reported subjective symptoms, and received medications, hence it is the least objective parameter used to qualify for CRT and it is responsible for large variations in the number of patients who could receive CRT. The presence of LBBB, LVEF value, QRS width, and heart rhythm allow for greater objectivization of indications for CRT.

QRS duration predicts CRT response and was the inclusion criterion in all randomized clinical trials. Since 2016, the ESC guidelines based on the Echo-CRT trial did not recommend CRT in patients with a QRS duration < 130 ms, due to suggested possible harm from CRT in these patients [1,3,36,37]. According to the AHA/ACC/HFSA 2022 guidelines, CRT implantation is possible with a QRS duration ≥ 120 ms, both in LBBB and non-LBBB patients [7]. In our study, 3.2% of patients with SR and a QRS duration of 120–129 ms could have qualified for CRT following the AHA/ACC/HFSA 2022 criteria.

Several studies showed that LBBB morphology is more likely to have a favorable response to CRT when compared to non-LBBB morphology. CRT significantly reduced (36% reduction) a composite endpoint in patients with LBBB, while such benefits were not observed in patients with non-LBBB conduction abnormalities [38,39].

It should also be considered that due to the high complexity of the procedure, CRT implantation is associated with a significant risk of complications (including infectious and haemorrhagic), especially in patients with multiple comorbidities (including long-term diabetes, advanced chronic kidney disease, and cancer), in advanced age, or whose life expectancy does not significantly exceed one year. Hence, it is important to be familiarized with the currently applicable guidelines and to apply them in practice after taking into consideration the individual profile of a single patient. Current therapeutic strategies and risk assessment are imperfect as they are based in majority only on LVEF, NYHA, QRS width and LBBB presence, and do not include more specific criteria. Still more data, particularly from real-world patient studies, are necessary to improve and personalize HF management.

Real-world data suggest that even around 30% of the HFrEF population would have indications for CRT [1]. However, a recent survey from Sweden showed that of nearly 13,000 HFrEF patients, only 7% had received CRT, while 24% had an indication but had not received CRT [40]. QUALIFY (QUAlity of adherence to guideline recommendations for LIFe-saving treatment in heart failure: an international surveY) showed a low use of implantable devices in patients with HFrEF: CRT-D (6.3%) and CRT-P (1.4%) [41]. In the previous analyses of the ESC-HF Pilot and ESC-HF Long-Term, CRT was present in 1.8% and 5.9% of patients, respectively [42,43]. In our study, the biggest number of patients would be eligible for CRT according to the 2013 ESC guidelines (18.3%), whereas based on the recent ESC 2021 and AHA/ACC/HFSA 2022 guidelines, only 12.7% and 17.6% were eligible for CRT, respectively. These results indicate that there are significant differences in the eligibility criteria between the current guidelines. Regardless of the guidelines, there seems to still be potential to increase the frequency of CRT use in patients with HF, as it should be pointed out that, with regard to the criteria, more patients could in fact receive CRT.

Our study possesses some limitations. The inclusion of real-life patients with HF followed up by cardiologists is an important advantage of ESC-HF Pilot and ESC-HF-LT registries, but drawbacks include the observational character. Furthermore, only the predefined data in the Case Report Forms designed by the coordinators of the registries were available for analysis (e.g., data on indications for permanent pacing, AVN ablation, and expected survival time were lacking). The registries were not primarily focused on the indications for CRT implantation, hence analyses errors are possible. The exact number of patients that would have effectively received CRT based on the guidelines is unknown. However, this study was designed to present a general concept of the differences in eligibility for CRT in patients with HF according to the recent international guidelines.

## 5. Conclusions

The results of our study show that a high variability in the percentage of patients meeting the CRT eligibility criteria was observed since 2007. Across the fifteen years, the criteria overlapped entirely only in a small percentage of cases. Despite general consistency in international guideline recommendations for CRT implantation, each time the decision to qualify a patient for CRT (and the choice between CRT-P and CRT-D) requires an individual approach and a careful assessment of indications and contraindications for this form of HF therapy.

## Figures and Tables

**Table 1:** Baseline characteristics of the study group.

Variable	HF Patients *n* = 1456
**Baseline characteristics**	
Age, years	66.5 (58.0–76.4)
Male	981 (67.4%)
BMI, kg/m^2^	27.8 (25.0–31.4); *n* = 1404
LVEF, %	37 (25–50)
LBBB QRS morphology	199 (13.7%)
QRS, ms	103 (90–120)
HFrEF	798 (54.8%)
HFmrEF	288 (19.8%)
HFpEF	370 (25.4%)
Previous HF hospitalization	781 (53.9%); *n* = 1450
Coronary artery disease	788 (54.2%); *n* = 1455
Moderate or severe mitral regurgitation	623 (45.9%); *n*= 1358
Hypertension	935 (64.3%); *n* = 1453
AF	442 (30.4%)
Peripheral artery disease	170 (11.7%); *n*= 1455
Diabetes	470 (32.3%)
Chronic kidney disease	260 (17.9%); *n* = 1454
COPD	236 (16.2%); *n* = 1454
Prior stroke or TIA	160 (11.0%); *n* = 1454
Current or former smoking	835 (58.0%); *n* = 1440
**Clinical status**	
Heart rate, bpm	70 (65–80); *n* = 1054
NYHA class	2 (2–3)
NYHA class I	138 (9.5%)
NYHA class II	894 (61.4%)
NYHA class III	400 (27.5%)
NYHA ambulatory class IV	24 (1.6%)
**Pharmacotherapy**	
ACE-I	1120 (77.0%); *n* = 1455
ARB	162 (11.1%); *n* = 1454
β-blocker	1317 (90.5%); *n* = 1455
Diuretic	1211 (83.2%); *n* = 1455
MRA	967 (66.5%); *n* = 1454
Statins	960 (66.0%); *n* = 1455
Oral Anticoagulant	606 (41.7%); *n* = 1454
Antiplatelets	902 (62.0%); *n* = 1455
Digitalis	336 (23.1%); *n* = 1455
Amiodarone	127 (8.7%); *n* = 1455
Other Antiarrhythmic	81 (5.6%); *n* = 1455
CCB	214 (14.7%); *n* = 1455

ACE-I—angiotensin-converting enzyme inhibitor; AF—atrial fibrillation; ARB—angiotensin receptor blocker; BMI—body mass index; bpm—beats per minute; CCB—calcium channel blocker; COPD—chronic obstructive pulmonary disease; LVEF—left ventricular ejection fraction; MRA—mineralocorticoid receptor antagonist; NYHA—New York Heart Association; HFmrEF—heart failure with mid-range ejection fraction; HFrEF—heart failure with reduced ejection fraction; HFpEF—heart failure with preserved ejection fraction; TIA—transient ischemic attack. Continuous variables are presented as medians and interquartile ranges.

**Table 2:** The changes in eligibility for CRT of real-life patients with heart failure according to differences in the last fifteen-years international guidelines.

Class *	Guidelines Listed by Year of Publication								*p*-Value
	2022 (ACC/ AHA/ HFSA)	2021 (ESC)	2016 (ESC)	2015 (ESC)	2013 (ESC)	2012/2013 (ACC/ AHA/ HRS)	2010 (ESC)	2007 (ESC)	
	**Patients with SR (*n* = 1014)**								
**I**	45 (4.4%)	45 (4.4%)	83 (8.2%)	53 (5.2%)	104 (10.3%)	45 (4.4%)	137 (13.5%)	98 (9.6%)	<0.001
**IIa**	97 (9.6%)	76 (7.5%)	38 (3.7%)	17 (1.7%)	35 (3.5%)	77 (7.6%)	-	-	<0.001
**IIb**	28 (2.8%)	33 (3.3%)	33 (3.3%)	28 (2.8%)	80 (7.9%)	48 (4.7%)	-	-	<0.001
	**Patients with AF (*n* = 442)**								
**IIa**	86 (19.5%)	31 (7.0%)	17 (3.8%)	17 (3.8%)	47 (10.6%)	54 (12.2%)	31 (7.0%)	47 (10.6%)	<0.001
	**Total (*n* = 1456)**								
	256 (17.6%)	185 (12.7%)	171 (11.7%)	115 (7.9%)	266 (18.3%)	224 (15.4%)	168 (11.5%)	145 (10.0%)	<0.001

ACC—American College of Cardiology; AHA—American Heart Association; AF—atrial fibrillation; ESC—European Society of Cardiology; HFSA—Heart Failure Society of America; HRS—Heart Rhythm Society; SR—sinus rhythm. * Class of recommendation: class I (indicated/recommended); class IIa (should be considered); class IIb (may be considered); class III (contraindicated/not recommended).

**Table 3:** Patients with heart failure and sinus rhythm eligible for CRT based on the recent ACC/AHA/HFSA (2022) and ESC (2021) guidelines.

Guidelines	SR OMT Ischemic Etiology	SR OMT LVEF ≤ 35%			
	**LBBB**				
	QRS ≥ 150 ms		QRS 130–149 ms		120–129 ms
	NYHA class I	NYHA class II–IV	NYHA class II	NYHA class III–IV	NYHA class II–IV
**ACC/AHA/HFSA 2022**	1 (0.1%)	45 (4.4%)	20 (2.0%)	18 (1.8%)	21 (2.1%)
**ESC 2021**	-	45 (4.4%)	20 (2.0%)	18 (1.8%)	-
	**Non-LBBB**				
	QRS ≥150 ms		QRS 130–149 ms		120–129 ms
		NYHA class II–IV	NYHA class II	NYHA class III–IV	NYHA class III–IV
**ACC/AHA/HFSA 2022**	-	38 (3.7%)	-	15 (1.5%)	12 (1.2%)
**ESC 2021**	-	38 (3.7%)	18 (1.8%)	15 (1.5%)	-

The meaning of colors: Green = class I of recommendation (indicated/recommended); Yellow = class IIa of recommendation (should be considered); Orange = class IIb of recommendation (may be considered); Red = class III of recommendation (contraindicated/not recommended); AHA—American Heart Association; ESC—European Society of Cardiology; HFSA—Heart Failure Society of America; LBBB—left bundle branch block; LVEF—left ventricular ejection fraction; NYHA—New York Heart Association; OMT—optimal medical treatment; SR—sinus rhythm.

**Table 4:** Patients with heart failure and atrial fibrillation eligible for CRT based on the recent ACC/AHA/HFSA (2022) and ESC (2021) guidelines.

Guidelines	ACC/AHA/HFSA 2022	ESC 2021
**Eligibility criteria**	AF Strategy to ensure biventricular capture LVEF ≤ 35%	
	QRS ≥ 120 ms	QRS ≥ 130 ms
	NYHA class II–IV	NYHA class III–IV
**Number of patients**	86 (19.5%)	31 (7.0%)

The meaning of color: Yellow = class IIa of recommendation (should be considered); AF—atrial fibrillation; AHA—American Heart Association; ESC—European Society of Cardiology; HFSA—Heart Failure Society of America; LVEF—left ventricular ejection fraction; NYHA—New York Heart Association.

## Data Availability

The data presented in this study are available for three years following the publication on request from the corresponding author.

## Supplementary Materials

The following supporting information can be downloaded at: https://www.mdpi.com/article/10.3390/jcm11113236/s1, Table S1: Indications for Cardiac Resynchronization Therapy in the major international guidelines.
