# Supplementary material for: Fifteen-Year Differences in Indications for Cardiac Resynchronization Therapy in International Guidelines—Insights from the Heart Failure Registries of the European Society of Cardiology
Source: J Clin Med. 2022 Jun 6;11(11):3236. doi: 10.3390/jcm11113236 (PMC9181415; doi:10.3390/jcm11113236)
Supplement: Supplementary file 1 [file jcm-11-03236-s001.zip › jcm-1738492-supplementary.pdf]

**Supplementary Table S1.** Indications for Cardiac Resynchronization Therapy in the major international guidelines.

| Guidelines | Class/LOE | Inclusion Criteria |
|---|---|---|
| **ESC 2007** | I/A<br>I/B | - NYHA class III-IV despite OMT<br>- LVEF ≤35%<br>- LV dilation*<br>- QRS ≥120 ms<br>- SR<br>*LV dilation (different criteria in clinical trials: LVEDD >55 mm or >30 mm/m2 or >30 mm/m (height)) |
| | IIa/C | - NYHA class III-IV despite OMT<br>- LVEF ≤35%<br>- LV dilation<br>- Indication for permanent pacing |

| | | |
|---|---|---|
| | IIa/C | - NYHA class III-IV despite OMT<br>- LVEF ≤35%<br>- LV dilation<br>- Permanent AF and indication for AV ablation |
| **ESC 2010** | I/A | - NYHA class III-IV** despite OMT<br>- LVEF ≤35%<br>- QRS ≥120 ms<br>- SR |
| | I/A | - NYHA class II despite OMT<br>- LVEF ≤35%<br>- QRS ≥150 ms<br>- SR |

| | | |
|---|---|---|
| | IIa/B | - NYHA class III-IV* despite OMT<br>- LVEF ≤35%<br>- QRS ≥130 ms<br>- Pacemaker dependency induced by AV nodal ablation |
| | IIa/C | - NYHA class III-IV* despite OMT<br>- LVEF ≤35%<br>- QRS ≥130 ms<br>- Slow ventricular rate and frequent pacing |
| **ACC/AHA/HRS 2012/2013** | I/A (B for NYHA class II) | - NYHA class II-IV** despite OMT<br>- LVEF ≤35%<br>- QRS ≥150 ms<br>- LBBB<br>- SR |

| | | |
|---|---|---|
| | IIa/B | - NYHA class II-IV** despite OMT<br>- LVEF ≤35%<br>- QRS 120-149 ms<br>- LBBB<br>- SR |
| | IIa/A | - NYHA class III-IV** despite OMT<br>- LVEF ≤35%<br>- QRS ≥150 ms<br>- Non-LBBB<br>- SR |
| | IIa/B | - NYHA class II-IV** despite OMT<br>- LVEF ≤35%<br>- LBBB QRS ≥120 ms lub non-LBBB QRS ≥150 ms<br>- Permanent AF and indication for AV ablation |

| | | |
|---|---|---|
| | IIb/C | - NYHA class I despite OMT<br>- LVEF ≤30%, ischemic etiology<br>- QRS ≥150 ms<br>- LBBB<br>- SR |
| | IIb/B | - NYHA class III-IV** despite OMT<br>- LVEF ≤35%<br>- QRS 120-149 ms<br>- Non-LBBB<br>- SR |
| | IIb/B | - NYHA class II despite OMT<br>- LVEF ≤35%<br>- QRS ≥150 ms<br>- Non-LBBB<br>- SR |

| Guidelines | Class/LOE | Inclusion Criteria |
| --- | --- | --- |
| **ESC 2013** | I/A | - NYHA class III-IV** despite OMT<br>- LVEF ≤35%<br>- QRS >150 ms<br>- LBBB<br>- SR |
| | I/B | - NYHA class II-IV** despite OMT<br>- LVEF ≤35%<br>- QRS 120-150 ms<br>- LBBB<br>- SR |
| | Iia/B | - NYHA class II-IV** despite OMT<br>- LVEF ≤35%<br>- QRS >150 ms<br>- non-LBBB<br>- SR |

| | | |
|---|---|---|
| | IIb/B | - NYHA class II-IV** despite OMT<br>- LVEF ≤35%<br>- QRS 120-150 ms<br>- non-LBBB<br>- SR |
| | IIa/B | - NYHA class III-IV* despite OMT<br>- LVEF ≤35%<br>- QRS ≥120 ms<br>- Permanent AF and indication for AV ablation |
| **ESC 2015** | I/A | - NYHA class III-IV despite OMT<br>- LVEF ≤35%<br>- QRS >150 ms<br>- LBBB<br>- SR |

| | | |
|---|---|---|
| | I/B | - NYHA class III-IV despite OMT<br>- LVEF ≤35%<br>- QRS 120-150 ms<br>- LBBB<br>- SR |
| | IIa/B | - NYHA class III-IV despite OMT<br>- LVEF ≤35%<br>- QRS >150 ms<br>- non-LBBB<br>- SR |
| | IIb/B | - NYHA class III-IV despite OMT<br>- LVEF ≤35%<br>- QRS 120-150 ms<br>- non-LBBB<br>- SR |

| | | |
|---|---|---|
| | IIa/B | - NYHA class III-IV despite OMT<br>- LVEF ≤35%<br>- QRS ≥120 ms<br>- non-LBBB<br>- Permanent AF and indication for AV ablation |
| **ESC 2016** | I/A | - NYHA class II-IV despite OMT<br>- LVEF ≤35%<br>- QRS ≥150 ms<br>- LBBB<br>- SR |
| | I/B | - NYHA class II-IV despite OMT<br>- LVEF ≤35%<br>- QRS 130-149 ms<br>- LBBB<br>- SR |

| | | |
|---|---|---|
| | IIa/B | - NYHA class II-IV despite OMT<br>- LVEF ≤35%<br>- QRS ≥150 ms<br>- non-LBBB<br>- SR |
| | IIb/B | - NYHA class II-IV despite OMT<br>- LVEF ≤35%<br>- QRS 130-149 ms<br>- non-LBBB<br>- SR |
| | IIa/B | - NYHA class III-IV despite OMT<br>- LVEF ≤35%<br>- QRS ≥130 ms<br>- non-LBBB<br>- Permanent AF and indication for AV ablation |

| | | |
|---|---|---|
| **ESC 2021** | I/A | - NYHA class II-IV despite OMT<br>- LVEF ≤35%<br>- QRS ≥150 ms<br>- LBBB<br>- SR |
| | IIa/B | - NYHA class II-IV despite OMT<br>- LVEF ≤35%<br>- QRS 130-149 ms<br>- LBBB<br>- SR |
| | IIa/B | - NYHA class II-IV despite OMT<br>- LVEF ≤35%<br>- QRS ≥150 ms<br>- non-LBBB<br>- SR |

| | | |
|---|---|---|
| | IIb/B | - NYHA class II-IV despite OMT<br>- LVEF ≤35%<br>- QRS 130-149 ms<br>- non-LBBB<br>- SR |
| | IIa/C | - Persistent or permanent AF<br>- NYHA class III-IV<br>- LVEF ≤35%<br>- QRS ≥130 ms<br>- Strategy to ensure biventricular capture in place |
| **AHA/ACC/HFSA 2022** | I/B-R | - NYHA class II-IV** despite OMT<br>- LVEF ≤35%<br>- QRS ≥150 ms<br>- LBBB<br>- SR |

| | | |
|---|---|---|
| | IIa/B-R | - NYHA class II-IV** despite OMT<br>- LVEF ≤35%<br>- QRS ≥150 ms<br>- non-LBBB<br>- SR |
| | IIa/B-NR | - NYHA class II-IV** despite OMT<br>- LVEF ≤35%<br>- QRS 120-149 ms<br>- LBBB<br>- SR |
| | IIb/B-NR | - NYHA class III-IV** despite OMT<br>- LVEF ≤35%<br>- QRS 120-149 ms<br>- non-LBBB<br>- SR |

| | | |
|---|---|---|
| | IIb/B-NR | - NYHA class I despite OMT<br>- Ischemic cause of HF<br>- LBBB<br>- QRS ≥150 ms<br>- SR |
| | IIa/B-NR | - AF<br>- LVEF ≤35%<br>- Patient requires ventricular pacing or otherwise meets the CRT criteria<br>- AV nodal ablation or pharmacological rate control will allow near 100% ventricular pacing with CRT |

**(NYHA class IV patients should be ambulatory = No admissions for HF during the last month and a reasonable expectation of survival >6 months)

The meaning of colors:

Green = class I of recommendation (indicated/ recommended)

Yellow = class IIa of recommendation (should be considered)

Orange = class IIb of recommendation (may be considered)

Red = class III of recommendation (contraindicated/ not recommended)

AF – atrial fibrillation; ACC – American College of Cardiology; AHA – American Heart Association; AV – atrioventricular; CRT – cardiac resynchronization therapy; LVEF – left ventricular ejection fraction; ESC – European Society of Cardiology; HF – heart failure; HFSA – Heart Failure Society of America; HRS – Heart Rhythm Society; LBBB – left bundle branch block; LOE – level of evidence; NYHA – New York Heart Association; OMT – optimal medical treatment; RV – right ventricle; SR – sinus rhythm
